# Foreign Correspondence

**Published:** 1887-11

**Authors:** Dudley C. Trott

**Affiliations:** M. B., F. R. C. S., Eng.; Hamilton, Bermuda


### FOREIGN CORRESPONDENCE.

To the Editor,

*Dear Sir:*—I have been requested, by some of your readers, to say a few words through your columns concerning Bermuda and the means of getting here.

Bermuda is situated in 32° north latitude and 65° west longitude, being about 700 miles from New York City.*

The Quebec Steam Ship Company sends good steamers every fortnight from Pier 47, North River, New York, leaving at 3 P. M. on Thursdays, and arriving here on the following Sunday morning. The fortnightly steamers are converted, on January 15th, into weekly steamers. During the month of April there will be a third steamer making several trips, in order that the return of visitors may be facilitated,—a matter which has heretofore prevented many from visiting the island.

Some individuals consider this a very sea-sick trip, but I am under the impression that, the voyage being so short, people have not time to get over *mal de mer* and enjoy it, whereas if the time were extended from 68 hours to six days, the end of this trip would be as pleasant as the end of most, and the mishaps of starting would be forgotten. The Steam Ship Company tells me it hopes in a year's time to reduce the sea trip to 52 hours, arriving on Saturday night instead of Sunday. The accommodations on board the boat are very good.

On the island there are two large hotels, the Hamilton and the Princess, the latter opening on December 10th, and the former on December 24th, and offering accommodations equal to the average first-class American resort houses, and at rates about equally current among them. There are, besides, numerous pleasant and comfortable boarding houses in various parts of the island, quotable at from $12 to $14 per week.

Temperature. — During the season proper, from December to May, the average minimum (for ten years) ranges from 55° to 65° Fahr., and the average maximum from 68° to 76°—the extreme minimum in the most exposed places being 48°, occurring about 4 A. M; the extreme maximum being 80° (unusually) in those months, seldom reaching 90°, even in summer. The extreme low temperature in the usual dwelling parts is about 54°. From 9 A. M. to 5 P. M. the thermometer usually ranges from 60° to 70°, seldom varying more than 5° in one day.

The barometer may go to 29.°0 during a storm, but usually it remains at from 30.°0 to 30.°6. As is easily seen from the above temperature, patients with diseases requiring an even temperature will find that here. Among those diseases which a residence in Bermuda may benefit are:

1. Genito-urinary troubles.
2. Chronic rheumatism and gout.
3. Bronchitis, chronic throat affections and fibroid phthisis.
4. Phthisis in its earliest stages, limited to cases without loss of lung-tissue, such as are benefited by a prolonged sea

* Messrs. A. E. Outerbridge & Co., of 51 Broadway, New York, agents of the Quebec Steam Ship Company, can give information that may be desired.

voyage with the even temperature (limited consolidations, early hæmorrhages, prolonged respiration). Cases with cavities in the lungs should not be sent here. In early cases of phthisis our climate acts as it does in bronchitis. My experience is that early cases are usually benefited here, provided the patients observe the ordinary precautions of avoiding the night air as much as possible. From 9 A. M. until 5 P. M. patients can usually be out, driving, yachting or walking, the air seldom being either chilly or too warm for sufficient exercise. I have frequently seen those who have had a few hæmorrhages continue well as long as they followed the practice of spending the winter here.

5. Rest, in cases of nervous prostration and of over-work, can be had here probably more absolutely than elsewhere, as there is no cable, and business communications and prices of stocks can only arrive in weekly installments.

6. MALARIA.—As we have no fresh water swamps we are absolutely free from malarial miasma, our soil and rock being so porous that water rapidly runs from the surface.

7. Convalescents from acute and other affections.

8. Dyspepsia.

I need hardly mention that, for well people, who wish to avoid the cold and who can do with a limited amount of excitement, as well as for children, Bermuda is charming.

DUDLEY C. TROTT, M. B.,
F. R. C. S., Eng.

HAMILTON, BERMUDA,

November 1, 1887.

